# CX3CL1 reduces neurotoxicity and microglial activation in a rat model of Parkinson's disease

**DOI:** 10.1186/1742-2094-8-9

**Published:** 2011-01-25

**Authors:** Mibel M Pabon, Adam D Bachstetter, Charles E Hudson, Carmelina Gemma, Paula C Bickford

**Affiliations:** 1 Department of Molecular Pharmacology and Physiology, University of South Florida, College of Medicine, Tampa, FL 33612, USA; 2 Department of Neurosurgery, Center of Excellence for Aging and Brain Repair, University of South Florida, College of Medicine, Tampa, FL 33612, USA; 3 Sanders-Brown Center on Aging, University of Kentucky, Lexington, KY 40536 USA; 4 James A. Haley Veterans Administration Hospital, Tampa, FL 33612, USA

## Abstract

**Background:**

Parkinson's disease is characterized by a progressive loss of dopaminergic neurons in the substantia nigra. The cause of the neurodegeneration is unknown. Neuroinflammation has been clearly shown in Parkinson's disease and may be involved in the progressive nature of the disease. Microglia are capable of producing neuronal damage through the production of bioactive molecules such as cytokines, as well as reactive oxygen species (ROS), and nitric oxide (NO). The inflammatory response in the brain is tightly regulated at multiple levels. One form of immune regulation occurs via neurons. Fractalkine (CX3CL1), produced by neurons, suppresses the activation of microglia. CX3CL1 is constitutively expressed. It is not known if addition of exogenous CX3CL1 beyond otherwise physiologically normal levels could decrease microglia activation and thereby minimize the secondary neurodegeration following a neurotoxic insult.

**Methods:**

The intrastriatal 6-hydroxydopamine (6-OHDA) rat model of Parkinson disease, was used to test the hypothesis that exogenous CX3CL1 could be neuroprotective. Treatment with recombinant CX3CL1 was delivered to the striatum by an osmotic minipump for 28 days beginning 7 days after the initial insult. Unbiased stereological methods were used to quantify the lesion size in the striatum, the amount of neuronal loss in the substantia nigra, and the amount of microglia activation.

**Results:**

As hypothesized, CX3CL1 was able to suppress this microglia activation. The reduced microglia activation was found to be neuroprotective as the CX3CL1 treated rats had a smaller lesion volume in the striatum and importantly significantly fewer neurons were lost in the CX3CL1 treated rats.

**Conclusion:**

These findings demonstrated that CX3CL1 plays a neuroprotective role in 6-OHDA-induced dopaminergic lesion and it might be an effective therapeutic target for many neurodegenerative diseases, including Parkinson disease and Alzheimer disease, where inflammation plays an important role.

## Background

Parkinson's disease (PD) is a neurodegenerative disorder affecting the motor system including motor coordination and speed as well as producing rigidity and tremor. The symptoms of PD are mainly due to a progressive loss of dopaminergic neurons within the pars compacta of the substantia nigra (SNpc). This degeneration decreases the levels of the neurotransmitter dopamine in the nigrostriatal system. In the past 15 years, an increasing amount of evidence has emerged to suggest that inflammation may contribute to nigrostriatal pathway degeneration and accelerate the progression of pathology in PD patients. Within the microenvironment of the brain, glial cells play a critical role in homeostatic mechanisms that promote neuronal survival. There is a long history demonstrating, at least a casual link, in humans between microglia activation and PD, including early studies, which showed activated microglia in the substantia nigra of PD patients and people exposed to MPTP [1,2]. The contribution of microglia activation to the degenerative process in PD is presently unknown, but is likely to have a damaging rather than a beneficial role [3].

In animal models that mimic many aspects of Parkinson disease, activated microglia have been shown capable of damaging and killing neurons (For reviews on the topic see: [3,4]). However microglia do not typically kill neurons unless they are activated by a 'danger signal' such as the release of ATP, DNA, or mitochondria from a cell, which can occur when a cell dies and is not efficiently cleared [5]. While 'danger signals' can activate microglia there are also a number of suppressive signals that neurons use to induce 'immune tolerance' to protect the neurons from being killed. Recent evidence indicates that neurons are not only passive targets of microglia but rather can control microglial activity. Fractalkine (CX3CL1) is one of the signals that neurons constitutively express that plays a 'calming' role to reduce microglial activation by ligation of CX3CL1 to a G-protein coupled receptor (CX3CR1) present on microglia. CX3CL1 is a transmembrane chemokine and exists in both membrane-bound and soluble forms. Its membrane-bound form displays adhesion properties and consists of an intracellular domain and a transmembrane domain [6,7]. Soluble CX3CL1 form results from the cleavage of membrane-bound CX3CL1 by the metalloproteinase ADAM 10 and ADAM 17 [8,9].

In contrast to many other chemokines, CX3CL1 binds to only one receptor (CX3CR1). This receptor responds to membrane-bound CX3CL1 and to the soluble form. CX3CL1 acts *in vitro *as an anti-inflammatory molecule by down regulating IL-1β, TNF-α and, IL-6 production [10,11]. CX3CL1 has an essential role in protecting the brain from a dysregulated microglial response that leads to neurotoxicity [12]. Yet, while it is clear that complete loss of CX3CL1 signaling leaves neurons susceptible to microglia mediated neuronal injury and death, however it is not clear if supraphysiological levels of CX3CL1 would be neuroprotective. CX3CL1 and the CX3CL1 receptor are normally expressed at relatively high levels in the brain [12,13]. It has been shown that there are age related changes in CX3CL1 [14,15], but there is not a complete loss. Moreover, it is not known, at normal physiological conditions, if CX3CL1 or the CX3CR1 are in excess. As this signaling pathway is very important for regulating inflammation in the brain, it is likely that the ligand would be in excess to ensure tight regulation of the immune response. When neurons are lost there will be changes to the levels of CX3CL1. Surrounding 'healthy' neurons could be left vulnerable to the neuroinflammatory response due to the altered local CX3CL1 changes. Therefore, this study sought to determine if the CX3CL1 pathway could be a therapeutic target to prevent excessive microglia activation that contributes to neurodegenerative disease in the 6-OHDA, toxin induced, model of Parkinson's disease. The results of this study demonstrate that even small increases in CX3CL1 can be neuroprotective, by suppressing microglia activation.

## Methods

### Animals

All experiments were conducted in accordance with the National Institute of Health Guide and Use of Laboratory Animals, and were approved by the Institutional Animal Care and Use committee of the University of South Florida, College of Medicine. 3 months old male Fisher 344 (F344) rats (Harlan Sprague Dawley, Indianapolis, IN), were pair-housed in environmentally controlled conditions (12:12 h light:dark cycle at 21 ± 1°C) and provided food and water *ad libitum. *Animals were excluded from the study if they developed post-surgery infection, the osmotic pump or cannula was dislodged, or placement of the cannula was not at specified coordinates on post-mortem evaluation.

### Surgical Procedures

All surgical procedures, followed previously described methods [14,16]. Rats were anaesthetized with isofluorane. Depth of anesthesia was monitored and adjusted as necessary. All procedures were performed using sterile techniques. Briefly, the head was incised using a sterile scalpel blade; bleeding was minimized using cotton swabs. A hole was drilled for placement of a skull screw to be used as anchors for dental acrylic. A second hole was drilled over the dorsal striatum for placement of a cannula at the following coordinates: AP = +1.0 mm; ML = +3.0 mm and V = -4.5 mm. 6-OHDA (20 μg/4 μL) was infused at a rate of 0.5 μL per minute into the left striatum (PMID: 17346684). Immediately after the 6-OHDA lesion a stereotactically implanted cannula (coordinates: AP = +1.0 mm; ML = +3.0 mm and V = -4.5 mm) was affixed to the skull by dental acrylic and attached to and osmotic minipump inserted subcutaneously, for a sustained delivery of saline for 7 days. After the first 7 days, a mid-scapular incision was made and the saline pump was switched for the treatment pump to deliver 3 ng/day (n = 5), 30 ng/day (n = 4), or 90 ng/day (n = 5) of CX3CL1 or 90 ng/day of heat-inactivated fractalkine (n = 6) (as a control) for 28 days into the site of the lesion (*Alzet model 2004: pumping rate 0.25 μl/hr (± 0.05 μl/hr); total volume 1.0 mL*) as previously described [14]. After day 28 the rats were anesthetized and perfused transcardially with phosphate buffered saline (PBS) followed by 4% paraformaldehyde. Brains were then removed and postfixed in paraformaldehyde overnight; they were then transferred to a 30% sucrose/PBS solution for at least 16 hours. Coronal sections were cut (40 μm) using a cryostat and the sections were collected and stored in a cryoprotectant solution at -20° until processing.

### Immunohistochemistry

Tyrosine hydroxylase (TH) immunohistochemical staining was performed on free floating sections on every third sections for the striatum and every sixth section for the entire substantia nigra. With a random start, and including sections before and after both anatomical regions to make sure the entire structure was quantified. Using standard staining procedure the sections were pretreated with tris buffered saline/NaIO4 (sodium periodate) for 20 minutes at RT (60 rpm) to block endogenous peroxidase activity. Then washed with 0.1 M PBS (pH 7.2-7.5). Tissue was then blocked with a PBS/0.3% triton X-100/10% serum (Normal Horse serum; Lampire Biological Labs; cat # 7333400) mixture for one hour at room temperature. Sections were then incubated with the primary antibody (mouse anti-TH, 1:10,000; Immunostar cat# 22941) diluted into 3% horse serum with 0.3% Triton X-100 overnight at 4°C. The following day the sections were washed and then incubated for one hour with a biotinylated horse anti-mouse as secondary antibody (Vector cat# BA-2001) at a concentration of 1:1000 in PBS-TS for an hour at room temperature. The secondary antibody was amplified using avidin-biotin substrate (ABC solution, Vector Cat# PK-6100) for 1 hour at room temperature. Finally the sections were developed with 3,3'-Diaminobenzidine tetra-hydrochloride (SIGMA FAST ™ DAB). Sections were then mounted onto glass slides and dried overnight. The next day slides were pass trough a gradient of ethyl alcohol and xylene to dehydrate the tissue. The slides were then coverslipped using permount mounting medium (Thermo Fisher Scientific Inc. Cat. # SP15-500).

OX-6, a marker for MHCII positive microglia, immunohistochemical staining was performed on free-floating sections following standard staining procedures with the following adjustments: One in every third section was stained including beginning to end of the striatum and one in every six section was used beginning to end of the substantia nigra. Sections were washed three times in PBS, then tissue endogenous peroxidase activity was quenched using 0.3% H_2_O_2 _solution combined with 40% methanol for 20 min preceded by washing with PBS three times. Tissue was then blocked with 10% normal horse serum and 0.3% Triton X-100. Monoclonal antibody was used as primary antibody directed against the rat major histocompatibility II (mouse anti-RT1B; BD cat# 554926) at a concentration of 1:750 into PBS-TS solution overnight at 4°C. On day 2 of this protocol, tissue was washed three times with a solution of PBS enhanced with 3% horse serum. Sections were then incubated with biotinylated secondary antibody (Horse anti-mouse with a concentration of 1:300 from Vector cat # BA-2001) for an hour at room temperature. The staining proceeded as described for TH above with the exception that the DAB used contained metal enhancer (Vector Cat# SK-4100).

NeuN immunohistochemistry was performed as previously mentioned following the standard staining procedures to stain one in sixth free floating sections of the substantia nigra. With the following alterations: To quench endogenous peroxidase a solution of 40% methanol/2% H_2_O_2 _was used. Additional washes proceded by a one hour incubation with blocking solution of 10% horse serum and 0.3% Triton X-100. Primary antibody incubation was performed using mouse anti-NeuN (Millipore Cat# 3777) with a concentration of 1:1000 diluted into PBS-TS solution overnight at 4°C. On the following day of this protocol tissue was washed three times with PBS enhanced with 3% horse serum. Sections were then incubated with a biotinylated secondary antibody horse anti-mouse (from Vector cat # BA-2001) at a concentration of 1:300 for an hour at RT in PBS-TS. The staining continued as indicated above for TH staining.

### Stereological Quantification

Stereological methods were used for quantification of cells present in the tissue stained as previously described [14,16]. Briefly, tissue was viewed with an Olympus BX60 microscope and MicroBrightField, CX 9000 camera. The tissue was quantified using optical fractionator from MicroBrightField, software of Stereo Investigator (Ver.8). The estimated volume (μm^3^) of TH negative zones was quantified using cavalieri method of unbiased stereology in the striatum of every third section. Also, the expression of OX-6 positive cells was quantified in the striatum through the use of cavalieri. Both staining were quantified using a grid spacing of 200 μm using a 2x/0.06 objective. TH positive cells are quantified within the area of the substantia nigra pars compacta. The sampling site is customized to count 200 cells per brain sampling was with error coefficients less than 0.07. For counting TH positive cells the counting frame (CF) is 70 × 70 with a virtual counting grid (CG) of 140 × 140. For OX-6 cells CF is 400 × 300 and CG of 400 × 300. For NeuN stain CF is 75 × 75 and CG is 160 × 160.

### Statistical Analysis

Data are presented as mean cell number ± SEM averaged across subjects per group, or mean volume ± SEM averaged across subjects. Statistical comparison of the data was performed using GraphPad Prism version 5.00 for Windows (GraphPad Software, San Diego California USA, http://www.graphpad.com). Group interaction was analyzed with one-way ANOVA, followed by Tukey post-hoc analysis.

## Results

### CX3CL1 protects against 6-OHDA induces dopamine cell loss and neurodegeneration

**Figure 1:**
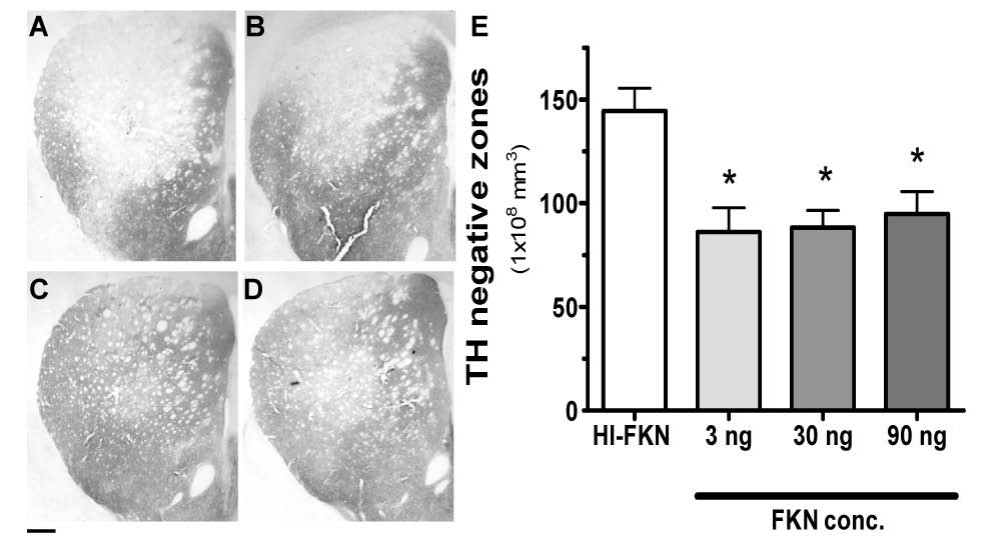
**CX3CL1 reduces 6-OHDA induced lesion volume in striatum**: photomicrographs representing TH immunohistochemistry following a 6-OHDA lesion. In the dorsal striatum a noticeable decrease of TH immunoreactivity is seen in the control group (HI CX3CL1; A) as compared to the groups who received exogenous CX3CL1 for 28 days; 3 ng (B), 30 ng (C) CX3CL1 90 ng (D). Bar denotes 0.5 mm. Quantification of the TH negative zone in the striatum of 3-month-old rats as determined by the Cavalieri method of unbiased stereology, demonstrates significant decrease in the TH negative immunoreactivity when CX3CL1 was administered at the different concentrations (3, 30, and 90 ng) for 28 days (E). The group that received HI-CX3CL1 had a significant larger lesion size. These findings indicate that CX3CL1 plays a neuroprotective role in the 6-OHDA model of Parkinson disease. One way ANOVA testing [F_(3,19) _= 7.149, p = 0.0029]. Asterisk denotes significance (* P < 0.05) of Tukey *post-hoc *analysis compare to HI-CX3CL1 group.

Intrastriatal 6-OHDA induces loss of dopamine terminals in the striatum and cell loss in the SNpc and is used as model of PD like neurodegeneration. Figure 1 shows the loss of the TH immunoreactivity in the striatum of rats 5 weeks after an injection of 6-OHDA. PD is a slowly progressing neurodegenerative disease, which requires substantial cell loss of the substantia nigra to occur before clinical symptoms are evident. To determine if the CX3CL1 signaling pathway could be protective during the progressive degenerative phase of the disease we began a sustained intrastriatal infusion by osmotic minipump of CX3CL1 beginning one week after the initial insult of 6-OHDA and lasting for four weeks. Three doses of CX3CL1 (3, 30, or 90 ng/day) were tested in this model and compared to treatment with heat inactivated (HI)-CX3CL1. As shown in Figure 1, all three doses of CX3CL1 resulted in a marked protection of the TH^+ ^terminals in the striatum. Quantification of the amount TH^+ ^terminals loss showed that all three concentration of CX3CL1 resulted in a similar 50% reduction in the size of the TH negative zone (Figure 1.E). The amelioration of the lesion size by CX3CL1 was significant for all three doses. There was no difference between the different doses in the ability to protect the terminal from degeneration.

### CX3CL1 suppress the activation of microglia in the striatum following a 6-OHDA insult

**Figure 2:**
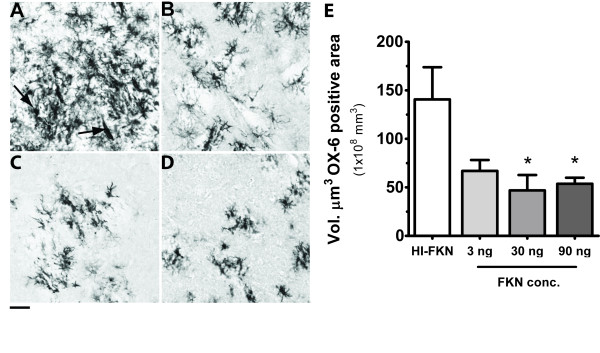
**CX3CL1 suppresses microglia activation**: Micrographs of OX-6 positive cells (MHCII; a marker for actived microglia) show a large increase in OX-6 staining in the control treated animals 5 weeks after a 6-OHDA in to striatum (A). Twenty-eight days infusion of CX3CL1 3 ng (B), CX3CL1 30 ng (C), or CX3CL1 90 ng (D), beginning 7 days after the insult with 6-OHDA show a clear reduction in OX-6 staining. Bar denotes 0.5 mm. Arrow denotes ameboid microglial cell (Panel A). Quantification of OX-6 immunoreactivity, by the Cavalieri method, found a significant decrease in OX-6 expression when exogenous CX3CL1 was administered (30 ng and 90 ng) as compared to the heat inactivated control (E). One way ANOVA testing [F_(3,19) _= 4.172, p = 0.0232]. Asterisk denotes significance (* P < 0.05) of Tukey *post-hoc *analysis compare to HI-CX3CL1 group.

Signaling by CX3CL1 to the only known receptor, CX3CR1, has been shown to be critical for restraining microglia activation. Moreover, in the CNS, CX3CR1 is only expressed on microglia [12,13,17]. The CX3CL1effects demonstrated by CX3CL1 in protecting the TH^+ ^terminals were hypothesized to occur by suppression of microglia activation. The major histocompatibility complex II (MHCII) is normally undetectable on microglia. Activation of the microglia induces MHCII expression, and presumably indicates a detrimental form of microglia activation. To assess the activation of microglia we stained for MHCII expression using the antibody OX-6. As shown in figure 2, at 5 weeks after the 6-OHDA insult there was a large volume of OX-6 positive staining in the striatum of the HI-CX3CL1 treated rats. As can be observed in figure 2B-C, there was a reduction in MHCII staining in the CX3CL1 treated animals. Using cavalieri method to assess the volume of MHCII expression we compared the effect of CX3CL1 in the STR in all of the groups (Figure 2E). A significant decrease in the volume of OX-6 positive cells was found in the rats treated with 30 or 90 ng/day of CX3CL1.

### Neuronal loss and microglia activation in the substantia nigra is reduced by treatment with CX3CL1

**Figure 3:**
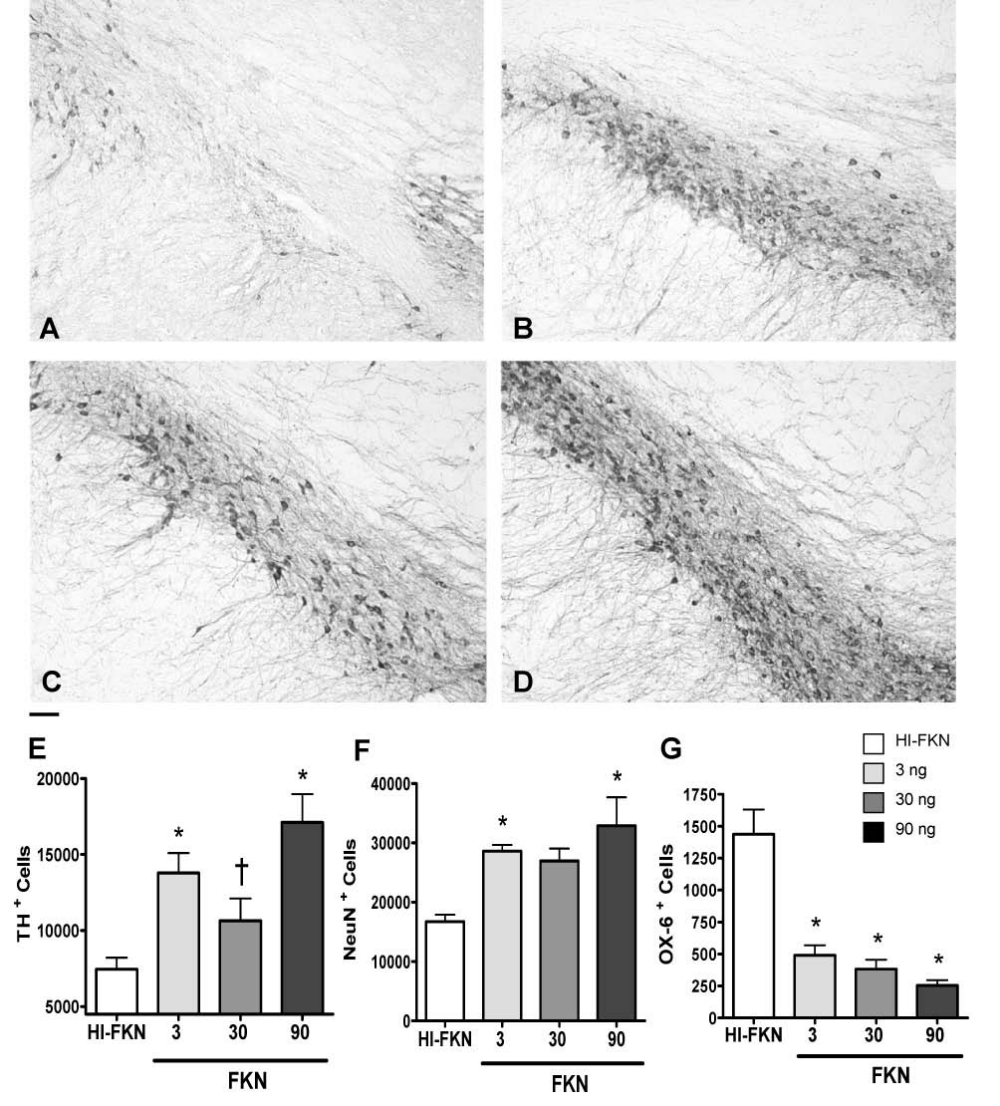
**Neurodegeneration is prevented in the substantia nigra**. Photomicrographs of TH immunoreactivity in substantia nigra demonstrate a noticeable decrease in TH immunoreactivity in the control groups (A) as compared to the groups that received 3 ng (B), 30 ng (C) and 90 ng of CX3CL1 (D). Bar denotes 0.2 mm. (E) Quantification of the number of TH^+ ^cells in the substantia nigra shows a significant decrease of TH immunoreactivity in the substantia nigra of animals who receive HI CX3CL1 (control treatment) but this affect was reversed when animals received 3, and 90 ng exogenous soluble CX3CL1 through and osmotic minipump for 28 days one week after a lesion with 6-OHDA. One-way ANOVA, [F_(3,19) _= 9.498, p = 0.0008]. Asterisk denotes significance (* P < 0.05) compare to HI-CX3CL1 group; † denotes significance († P < 0.05) compare to 90 ng CX3CL1 group as determined by Tukey *post-hoc *analysis. (F) Quantification of NeuN (neuronal nuclei marker) immunohistochemistry in substantia nigra shows a significant decrease in the HI-CX3CL1 control group as compared to the groups of animals treated with 3ng and 90 ng of CX3CL1. One-way ANOVA, [F_(3,19) _= 7.359, p = 0.0026]. Asterisk denotes significance (* P < 0.05) of Tukey *post-hoc *analysis compare to HI-CX3CL1 group. (G) An increase in the number of OX-6^+ ^cells in substantia nigra was found in the HI-CX3CL1 control group. CX3CL1This was significantly decreased by all three doses of CX3CL1. One-way ANOVA, [F_(3,19) _= 19.04, P < 0.0001]. Asterisk denotes significance (* P < 0.05) compare to HI-CX3CL1 group by Tukey *post-hoc *analysis.

To assess if this increased TH fiber density in the striatum reflected a protection from cell death in the substantia nigra TH positive cells were counted in the SNpc. Treatment with CX3CL1 at 3, 30, and 90 ng of CX3CL1 resulted in significant protection from loss of TH^+ ^cells compared with HI-CX3CL1 (Figure 3E). To confirm that the loss of TH^+ ^cells reflected cell death, NeuN immunoreactive cells were also counted in the SNpc (Figure 3F). The number of NeuN cells confirmed the results observed with TH^+ ^immunohistochemistry that the administration of exogenous soluble CX3CL1 helped protect the cell loss in the substantia nigra. While CX3CL1 was delivered directly into the striatum, protection of the terminals in the striatum could result in decreased microglia activation in the substantia nigra as a secondary interaction of less dying neurons. Quantification of the number of OX-6 positive cells in the SNpc confirmed this hypothesis (Figure 3G), and indicates that blocking inflammation in the striatum can decrease the inflammatory cascade in the substantia nigra.

## Discussion

This study sought to determine if the CX3CL1/CX3CR1 axis would be amendable to therapeutic intervention in a disease relevant animal model with a known inflammatory component as part of the neurodegeneration. The results of this study lend support to an important role of CX3CL1 in maintaining immune tolerance in the CNS. Following treatment with CX3CL1 neuronal protection was found that correlated with a reduction in microglia activation. Since microglia are the predominant cells in the CNS that express the CX3CL1 receptor, the effects of CX3CL1 are most likely a direct effect on microglia activity [12,13,17]. The state of microglia was assessed by the expression of MHCII, which is an often-used maker of a classical (M1) proinflammatory state of activation. However, it should be noted that in this study we can only infer from the changes in MHCII expression that CX3CL1 exerted the effects by reducing inflammation, as no direct cytokine measurements such as levels of IL-1β or iNOS were made. CX3CL1/CX3CR1 is important for suppressing the production of TNFα, IL-1β, IL-6 and INOS [11,12,18]; therefore, this effect of CX3CL1 to reduce cytokines and alter microglial phenotype away from M1 is the most likely interpretation of the data. There is a possibility that cannot be excluded from the results of this study, that exogenous CX3CL1 is having effects on cells other than microglia. CX3CR1 is expressed on a subset of dendritic cells and natural killer cells, peripheral blood monocytes and tissue macrophages [19]. *In vivo *neurons do not appear to have detectable levels of CX3CR1 [12,17], so while effects on other subsets of immune cells can not be ruled out, it is very unlikely that there is a direct effect of CX3CL1 of neurons.

An unexpected finding of this study was the lack of a dose response. A slight dose response was seen in some of the measurements, but a significant difference between the different treatment doses was not found. It is possible that the range of doses of CX3CL1 chosen in this study were too narrow to demonstrate a dose response. A second possibility for the lack of the dose response is that this signaling pathway, which is tonically activated during normal physiological conditions, has only a limited extra capacity to suppress glia activation. Therefore at doses above 3ng/day of CX3CL1 there is an excess of CX3CL1 to the amount of receptor available. It has been previously shown that by 7 days after a MPP^+ ^lesion in rats there is an increase in CX3CR1 protein levels in the substantia nigra; however, similar quantification was not made of CX3CL1 [20]. It is therefore unknown if a commensurate change in CX3CL1 is also found. Our results would suggest there is an increase CX3CR1 expression without a parallel increase in CX3CL1, leading to an imbalance in the CX3CR1/CX3CL1 axis. This imbalance is akin to what is seen in the CX3CR1 KO mice where disruption in the CX3CR1/CX3CL1 axis leads to neurotoxic inflammation [12].

A final possibility for the lack of a dose response could be due to a floor affect. Such that, 50% of the lesion occurs by 7 days before CX3CL1 treatment began, and this primary neurodegeneration cannot be reversed by CX3CL1 treatment; however, further neurodegeneration can be blocked by CX3CL1 treatment. We have previously observed in this model that there is a late phase of microglial activation that continues to increase from 1 to 4 weeks following the lesion and that if we block this late phase of inflammation we get neuroprotection; however, if we block the initial inflammation we can worsen neurodegeneration [16,21]. Nevertheless, all of the different concentrations of CX3CL1 showed neuroprotection in this Parkinson disease relevant model. This suggests that the communication between neurons and glial cells may play a role in Parkinson disease neurodegeneration.

Several reports have shown that CX3CL1 expression decreases during aging, and this decrease correlates with increased inflammation [14,15]. Consequently, changes in CX3CL1 could be important for the dysregulation of microglia in the age-related neurodegenerative disease. Interruption of the CX3CL1/CX3CR1 axis by genetic deletion causes neurodegeneration following stimulation with lipopolysaccharide, MPTP, or SOD1 mutation [12]. Finally, a large single-bolus of CX3CL1 injected into the substantia nigra has been shown to induce a Parkinson disease like symptoms or even cause death. However, it should be noted that the control for this injection of CX3CL1 was normal saline. It is therefore, unknown if the effects seen are due to the injection of a large amount of recombinant protein, the bovine serum albumin that was used as a carrier for the CX3CL1, or if the effect is indeed specific to CX3CL1 [20].

## Conclusions

In conclusion, this study demonstrates that the CX3CL1/CX3CR1 axis is an important target for drug discovery to modulate microglia activation in PD. Therefore, in the future it will be important to develop selective small-molecule agonists for CX3CR1 that are orally bioavailable and brain-penetrant. However, more work is also necessary to determine the role of CX3CL1 and CX3CR1 in normal physiological conditions, as well as, in models of neurodegenerative diseases.

## List of abbreviations

(CX3CL1): Fractalkine; (HI)-CX3CL1: heat inactivated; (6-OHDA): 6-hydroxydopamine;

## Competing interests

The authors declare that they have no competing interests.

## Authors' contributions

ADB, PCB, CG designed research. MMP, ADB, CEH, performed research. MMP, ADB wrote paper. All authors read and approved the final manuscript.
